# Bispecific antibody releasing-mesenchymal stromal cell machinery for retargeting T cells towards acute myeloid leukemia blasts

**DOI:** 10.1038/bcj.2015.73

**Published:** 2015-09-18

**Authors:** R Aliperta, M Cartellieri, A Feldmann, C Arndt, S Koristka, I Michalk, M von Bonin, A Ehninger, J Bachmann, G Ehninger, M Bornhäuser, M P Bachmann

**Affiliations:** 1 Department of Tumor-/Radioimmunology, Helmholtz-Zentrum Dresden-Rossendorf (HZDR), Institute of Radiopharmaceutical Cancer Research, Dresden, Germany; 2 University Cancer Center (UCC), Technical University Dresden, Tumorimmunology, Dresden, Germany; 3 Cellex Patient Treatment GmbH, Dresden, Germany; 4 Medical Clinic and Polyclinic I, University Hospital 'Carl Gustav Carus', Technical University Dresden, Dresden, Germany; 5 GEMoaB Monoclonals GmbH, Blasewitzer Strasse 41, Dresden, Germany; 6 DFG-Center for Regenerative Therapies Dresden, Technical University Dresden, Dresden, Germany

## Abstract

Bispecific antibodies (bsAbs) engaging T cells are emerging as a promising immunotherapeutic tool for the treatment of hematologic malignancies. Because their low molecular mass, bsAbs have short half-lives. To achieve clinical responses, they have to be infused into patients continously, for a long period of time. As a valid alternative we examined the use of mesenchymal stromal cells (MSCs) as autonomous cellular machines for the constant production of a recently described, fully humanized anti-CD33-anti-CD3 bsAb, which is capable of redirecting human T cells against CD33-expressing leukemic cells. The immortalized human MSC line SCP-1 was genetically modified into expressing bsAb at sufficient amounts to redirect T cells efficiently against CD33 presenting target cells, both *in vitro* and in an immunodeficient mouse model. Moreover, T cells of patients suffering from acute myeloid leukemia (AML) in blast crisis eliminated autologous leukemic cells in the presence of the bsAb secreting MSCs over time. The immune response against AML cells could be enhanced further by providing T cells an additional co-stimulus via the CD137-CD137 ligand axis through CD137L expression on MSCs. This study demonstrates that MSCs have the potential to be used as cellular production machines for bsAb-based tumor immunotherapy in the future.

## Introduction

T-cell engaging bispecific antibodies (bsAbs) are a promising tool for cancer treatment. This class of antibodies establishes a transient synapse between T cells and cancer cells by binding to a surface antigen on cancer cells with one arm and simultaneously recruiting T cells via the CD3 domain, which is the signal transmitting portion of the T-cell receptor complex.^1^ The polarization of the T-cell complex leads to an activation of bsAb recruited T cells and induces T-cell specific inflammatory and cytotoxic responses against the crosslinked target cells. A number of studies demonstrated that human primary T cells engaged with bsAbs lead to a profound anti-tumor reaction, both *in vitro* and *in vivo*.^2, 3^ During clinical trials with the CD19-CD3 specific bsAb blinatumomab, objective clinical responses were observed in most patients suffering from CD19-expressing non-Hodgkin's lymphoma and acute lymphoblastic leukemia.^4, 5^ In these studies the blinatumomab was delivered to patients by continuous intravenous infusion over several weeks. A continuous delivery of bsAbs seems to be necessary to sustain plasma levels in clinically relevant concentrations, as bsAbs have short half-lifes *in vivo* and are rapidly cleared from circulation due to their small molecule size.^6, 7^ An alternative to this approach, is the adoptive transfer of gene-modified cells, which produce and secrete bsAbs continuously in the body of the patient throughout their life-time. Due to their unique immunologic properties, human mesenchymal stromal cells (MSCs) seem to be a good choice for the generation of such cellular bsAb production machineries.^8, 9^ Experimental and clinical studies revealed that MSCs had limited immunogenicity and are even poorly recognized by HLA incompatible hosts.^10, 11, 12^ More importantly, MSCs tend to accumulate next to tumors, including metastatic lesions. Therefore, they can be used as a platform for the targeted delivery of anti-cancer agents.^13, 14, 15^ Furthermore, MSCs are appealing as cellular production machineries because they can easily be transduced with viral vectors, expanded *in vitro* and have a prolonged lifespan *in vivo*. On the other hand, studies showed that after an adoptive stem cell transplantation in patients with acute myeloide leukemia (AML), the transfer of MSCs provided an immunosuppressive environment, helpful to reduce graft versus host disease reactions.^16, 17^ This leads to the question, if an *in situ* production of bsAbs via MSCs interferes with the activation of bsAb redirected T lymphocytes.

In this study, for proof of concept, a recently described, fully humanized anti-CD33-anti-CD3 bsAb was chosen as therapeutic agent, which was to be produced by gene-modified MSCs.^2, 18, 19, 20, 21^ CD33 is predominantly found on the surface of myeloid-derived cells. In the bone marow of patients with AML, as well as in leukemic stem cells, it is overexpressed.^22, 23^ Depending on age and subtype of the disease, current, conventional AML therapies do not achieve long-term remissions. Therefore, new adjuvant therapeutic strategies are needed urgently, especially for the elimination of the minimal residual disease. Here we demonstrate that gene-modified MSCs are able to (i) express the CD33–CD3 specific bsAb at high levels and (ii) mediate an efficient lysis of AML blasts by human primary T cells of both healthy donors and AML patients.

## Materials and methods

### Ethics statement

Human peripheral blood mononuclear cells (PBMCs) were either isolated from buffy coats supplied by the German Red Cross (Dresden, Germany) or from fresh blood of healthy donors or from patients with their written consents. The study, including the consent form, was approved by the local ethics committee of the University Hospital of the medical faculty of the Carl-Gustav-Carus TU-Dresden (EK27022006).

NOD/SCID IL2Rγ^−/−^ mice were provided by the animal facility of the Technical University of Dresden. All procedures involving animals were performed according to the German animal protection law and with the permission of local authorities (Sächsische Landesdirektion).

### Cell lines

The human AML cell lines U937 (ACC 5) and MOLM-13 (ACC 554) were cultured in complete RPMI 1640 medium (Biochrom AG, Berlin, Germany). OCI-AML3 (ACC 582), HEK293T (ACC 635) and HEK293T-CD33 were cultured in complete DMEM medium.^19, 23^ The single-cell-picked clone 1 (SCP-1) cell line^24^ was grown in RPMI 1640 medium (10% FCS, 100 μg/ml penicillin/streptomycin). This cell line was previously derived from human MSCs and immortalized by lentiviral transduction using the gene coding for the human telomerase reverse transcriptase. Cell lines were maintained at 37 °C and 5% CO_2_.

### Generation of recombinant bsAb-releasing hMSCs

The development of the fully humanized anti-CD33-anti-CD3 bsAb was performed as previously described.^21^ For the generation of permanent hMSCs, releasing the bsAb, the complementary DNA, encoding the recombinant Ab construct, was cloned into the self-inactivating lentiviral vector p6NST50 to generate the transfer vector p6NST50.bsAb.EGFP-Zeocin.^25^ Lentiviral particles pseudotyped with the vesicular stomatitis virus envelope were generated by transient transfection of HEK293T cells.^26^ Virus supernatant was collected and used to stably transduce SCP-1 cells. For ectopic expression of the co-stimulatory 4-1BB ligand (CD137L) on the SCP-1 cell surface a lentiviral vector, harboring an internal minimal human elongation factor-1alpha promoter, was used.

### Flow cytometry analysis

Transduced and parental wild type (wt) cells were stained with anti-CD45/VioBlue, anti-CD33/PE, anti-CD90/VioBlue, anti-CD105/PE and anti-CD73/APC (all Miltenyi Biotec, Bergisch-Gladbach, Germany) monoclonal antibodies (mAbs) to analyze the hMSC marker profile. To monitor for transgenic 4-1BBL expression, SCP-1 cells were stained with an anti-CD137L/PE (BD Bioscience, Heidelberg, Germany) mAb. Samples were analyzed using a MACSQuant Analyzer and the MACSQuantify software (both Miltenyi Biotec).

### Expression, purification and quantitative analysis of the recombinant bsAb

The His-tagged anti-CD33-anti-CD3 bsAb released by gene-modified hMSCs, were purified from culture supernatants using single-step affinity chromatography on Ni-NTA columns (Qiagen, Hilden, Germany). They were analyzed by western blotting as previously described.^3, 27^ The amount of secreted anti-CD33-anti-CD3 bsAb was quantified by enzyme-linked immunosorbent assay (ELISA) as follows: F16 MaxiSorp ELISA plates were coated with 1.5 μg/ml mouse anti-pentaHis capture Ab (Qiagen), capable of binding the C-terminal histidine (his)-tag of CD33–CD3 bsAb. SCP-1 cells were seeded as triplets in 96-well plates at decreasing cell densities. After 48 h the supernatant of each sample was collected and added into the wells of the previously treated F16 MaxiSorp ELISA plates (Nunc, Rosklide, Denmark). The plates also contained a pre-defined dilution: standard samples were prepared as a two-fold serial dilution of the purified CD33–CD3 bsAb (GEMoaB, Dresden, Germany) starting from 100 ng/ml. For binding the captured bsAb, a detection solution (1:1000 dilution of anti-Myc-HRP detection Ab Miltenyi Biotec in blocking buffer) was added to the samples for 2 h at RT. The substrate solution, containing 100 μg/ml tetramethylbenzidine, 10% dimethyl sulfoxide, 0.05 M phosphate/citrate buffer and 30% H_2_O_2_, was added to each well. After 20 min the 1 m H_2_SO_4_ stop solution terminated the color reaction. Optical density at 450 nm, measured using the Sunrise Microplate Reader (Tecan, Maennedorf, Switzerland), was used to calculate the concentration of the hMSC-released bsAbs. Indirect immunofluorescence analysis was performed to examine the binding properties of the hMSC-released bsAbs. CD33^+^or CD33^-^ cell lines (5 × 10^5^) and PBMCs were stained with 20 μg/ml of the purified bsAbs and analyzed by flow cytometry using a FITC-conjugated antibody against the myc-tag (Miltenyi Biotec).^3, 27^ CD3^+^ T cells were discriminated using an anti-CD3/VioBlue mAb, whereas anti-CD56/APC (Miltenyi Biotec) and anti-16/PE (BD Biosciences, Heidelberg, Germany) mAbs were used to identify CD3^-^CD56^+^CD16^+^ natural killer cells. Maternal anti-CD33 and anti-CD3 mAbs, detected using a PE-conjugated Goat F(ab)_2_ anti-Mouse IgG (Fcgamma) Ab (Immunotech, Marseille, France), served as control.

### T cells isolation from healthy human donors

Isolation of human pan T cells and CD4^+^ and CD8^+^ T-cell subpopulations occurred from freshly isolated, human PBMCs of healthy, consenting volunteers using the pan T-cell or the CD4^+^ and CD8^+^ isolation kits (all Miltenyi Biotec). They were cultured in complete RPMI 1640 medium containing 50 U/ml IL-2 (ImmunoTools, Friesoythe, Germany).^3^

### ^51^Cr-release and flow cytometry-based cytotoxicity assay

The killing of CD33^+^ target tumor cells by T cells, redirected via hMSCs-released anti-CD33-anti-CD3 bsAb, was either examined by standard ^51^Cr release assays or by flow cytometry-based assays.^26, 27, 28^

### Determination of cytokine concentration

Cell-free supernatants were collected at the indicated time and analyzed for interferon-γ and tumor necrosis factor-α secretion, using the OptEIA ELISA Sets (BD Biosciences) according to the manufacturer's instruction.^3^

### T-cell activation and proliferation assays

To analyze the expression of the activation markers CD69 and CD25 on the surfaces of T cells, 1 × 10^4^ transgenic CD33-expressing HEK293T cells were co-cultured with 5 × 10^4^ untouched pan T cells in the presence or absence of 48 h cultured 1 × 10^4^ SCP-1 cells. At the indicated time points, triplicate cells were pooled and stained with a mixture of anti-CD3/VioBlue, anti-CD69/FITC, anti-CD4/PerCP (all purchased from Miltenyi Biotec) and anti-CD25/PE (BD Biosciences) mAbs. T-cell proliferation assays were performed as described.^25, 28^

### Flow cytometry killing assay with fresh AML samples

Upon approval by the local institutional review board, mononuclear cell (MNC) samples of consenting AML patients with hyperleukocytosis were prepared by gradient centrifugation over polydextran based separating solution Biocoll (Biochrom). The redirection of autologous T cells towards AML blasts by the bsAb-releasing hMSCs was investigated by co-cultivation of 1 × 10^4^ pre-cultured (48 h), genetically modified hMSCs with 1 × 10^5^ AML patient-derived MNCs. The specific killing of myeloid cells was analyzed with MACSQuant Analyzer (Miltenyi Biotec) at indicated time points. Moreover, one MNC per triplet was pooled and stained with a mixture of anti-CD3/PECy7, anti-CD4/PerCP mAbs (Miltenyi Biotec) to analyze the relative percentage of T cells and anti-HLA-DR/FITC and anti-CD45/VioBlue mAbs (Miltenyi Biotec) to discriminate the myeloid cell populations.

### Mouse model

NOD/SCID IL2Rγ^−/−^ mice were kept under standardized environmental conditions and received autoclaved food, water and bedding. Before the injection, bsAb-releasing or vector control containing hMSCs (5 × 10^3^) were cultured for 48 h and subsequently incubated with 1 × 10^5^ MOLM-13 cells and freshly isolated human T cells at an e:t ratio of 5:1. After 24 h of co-culturing, the mixed cell population was administrated intravenously (via the tail vein), into two groups of 8–10-week old NOD/SCID IL2Rγ^−/−^ mice. One group (five animals) received MOLM-13 cells, T cells and vector control hMSCs, whereas the other group (four animals) was treated with MOLM-13 cells, T cells and bsAb-secreting hMSCs. Mice were daily monitored for posture, activity, fur texture and skin integrity. Animals were sacrificed according to local ethical committee guidelines when displaying pathological scores and >15% body weight loss. The survival rate of bsAb-treated and untreated mice was determined.

### Statistical analysis

Statistical analysis was performed using the GraphPad Prism Software (GraphPad Software, Inc., La Jolla, CA, USA). One-way analysis of variance with the Bonferroni multiple comparison test were used for statistical significance when multiple experiments were compared. Survival data were analyzed using a Kaplan–Meier survival analysis with a log-rank method of statistics (***P<0.001, **P<0.01 and *P<0.05).

## Results

### Development and characterization of bispecific antibody-releasing hMSCs

**Figure 1:**
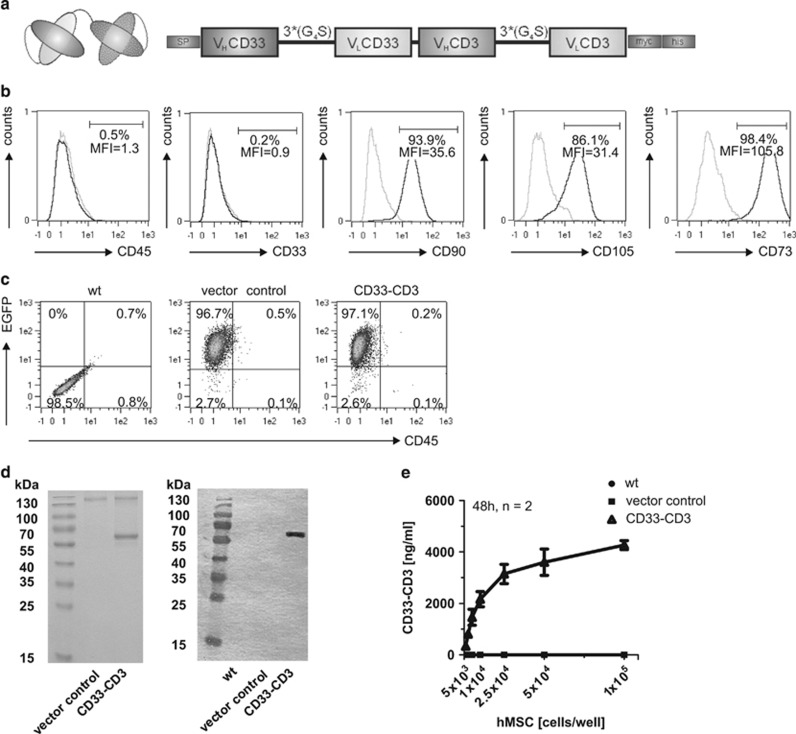
Generation of bsAb CD33–CD3-releasing gene-modified hMSCs. (**a**) Schematic representation of the structure of the bsAb CD33–CD3 constructed as single-chain bispecific tandem fragment variable (scBsTaFv). The V_H_ and V_L_ domains of each scFv were humanized by CDR grafting and connected via a linker comprised of three repeats of four glycine and one serine residues 3*(Gly_4_Ser). The N-terminus of the bsAb construct contains a signal peptide (SP) for the secretion of the bsAb into the cell culture medium, whereas its C-terminus tag harbors a myc- and his-tag used for immunochemical Ab detection and purification. (**b**) Flow cytometry analysis of the hMSC marker profile upon staining of the parental wild type SCP-1 line with VioBlue-, PE-, APC-, VioBlue- and PE-conjugated anti-CD90, anti-CD105, anti-CD73, anti-CD45, anti-CD33 mAbs (in black). Cells stained with matched isotype control Ab (in gray) served as negative control. Numbers represent the percentages of positive cells and the mean fluorescence intensity (MFI) of total cells. (**c**) Transgene expression analysis of parental and transduced hMSCs was performed by flow cytometry. The percentages of living CD45^-^ and EGFP^+^ cells are shown. Dead cells were excluded by propidium iodide staining. (**d**) Purified fractions of the bsAb CD33–CD3 secreted in the culture medium were separated on SDS-gels and thereafter stained either with Coomassie brilliant blue or analyzed by Western blotting. (**e**) The quantitative analysis of the released bsAb was performed by ELISA. hMSCs cells were seeded at limiting cell densities and the antibody concentration (ng/ml) in culture medium was determined after 48 h of culture. Results represent the means±s.d. of two independent experiments.

The hMSC lines were generated from the human telomerase reverse transcriptase-immortalized single-cell derived hMSC line SCP-1 and genetically modified using lentiviral gene transfer to stably express the bsAb CD33–CD3. The schematic representation of the anti-CD33-anti-CD3 bsAb is reported in Figure 1a. The parental SCP-1 cell line was examined for surface expression of the typical MSC marker proteins CD90, CD105 and CD73 and the absence of CD33, CD45 (Figure 1b) and CD34 (data not shown). For the selection of successfully transduced cells containing the *bsAb* gene, the *EGFP–zeocin* fusion gene was co-expressed under the same promoter through an internal ribosomal entry site. A lentiviral vector containing only the EGFP-zeocin expression cassette was used for the generation of a vector control cell line. The untransduced SCP-1 cell line served as wt control. After SCP-1 cell transduction each hMSC line (wt, vector control and CD33–CD3) was analyzed and selected for intracellular EGFP signal by flow cytometry (Figure 1c). Next, the secretion of the bsAb by gene-modified MSCs was verified. Therefore, culture medium was run over affinity chromatography columns to purify the bsAb CD33–CD3 through its C-terminal his-tag. Coomassie brilliant blue-staining and immunobloting analysis reveladed the presence of a ~60 kDa sized protein, which had the expected molecular size (Figure 1d). After successful bsAb production the amount of bsAb CD33–CD3 secreted by the modified MSCs was quantified by ELISA. At a starting density of 10^5^ hMSC cells/well, the maximum bsAb concentration was 4400 ng/ml. Considering a sample volume of 200 μl, a single MSC released ~8.8 pg bsAb in 48 h. The highest calculated Ab amount released by a single MSC (73.7 pg/cell in 48 h) was obtained at the lowest seeding density (10^3^ cells/well). The amount of released bsAb remains stable above 5 × 10^4^ cells/well (Figure 1e). Next, the binding specificity of the purified, MSC-released bsAb was analyzed: in agreement with previous analysis,^2, 18^ the binding of both maternal monoclonal antibodies lead to a strong shift in MFI (Supplementary Figure S1a). As expected the binding capabilities of the anti-CD33-anti-CD3 bsAb, was dependent on surface expression of the respective antigen, whereas no binding could be detected on antigen-negative cells such as HEK293T and SCP-1 (Supplementary Figure S1b). The bsAb showed a strong binding to the CD33 antigen, which can be blocked in a concentration-dependent manner using the maternal anti-CD33-specific mAb (Supplementary Figure S1c). In contrast, binding to the CD3 complex on T cells via the anti-CD3 domain was hardly detectable (Supplementary Figure S1a). The limited binding capability of the bsAb to the anti-CD3 domain represents a particular characteristic of the optimized bispecific antibody construct, required for a repeated rolling of the retargeted T cells and thus, a prerequisite for the optimal killing efficacy with lowest unspecific side effects.

### Specific CD33^+^ target cell killing by retargeted T cells via hMSCs-released bsAb

**Figure 2:**
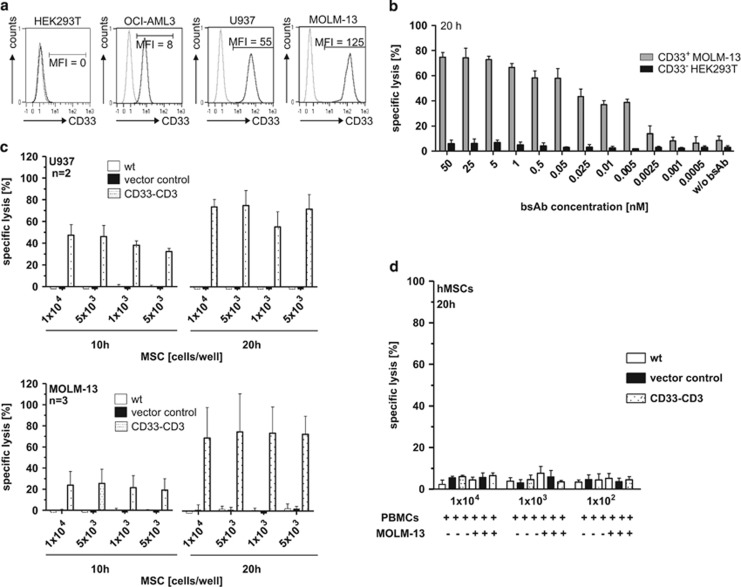
Efficient T-cell mediated killing of target cell lines with varying CD33 expression levels is induced in the presence of bsAb-releasing hMSCs. (**a**) HEK293T, OCI-AML3, U937 and MOLM-13 were analyzed for CD33 surface expression levels by staining with anti-CD33/PE mAb (in black) or matched isotype control Ab (in gray), respectively. Numbers represent mean fluorescence intensity (MFI) of total cells. (**b**) In a standard chromium release assay ^51^Cr labeled CD33^+^ MOLM-13 cells and CD33^-^ HEK293T cells were incubated with freshly isolated T cells at effector-to-target (e:t) cell ratio of 5:1 for 20 h with decreasing concentrations of the purified bsAb CD33–CD3. Mean±s.d. of two independent donors is shown. (**c**) Specific cell lysis of AML cell lines U937 (upper) and MOLM-13 (lower) measured with standard chromium release assay. Freshly isolated CD3^+^ T cells were co-cultured for 10 and 20 h with ^51^Cr labeled CD33^+^ target cells at an e:t cell ratio of 5:1 in the presence of hMSC lines seeded at different concentrations 48 h before adding effector T cells and target cells. Data are presented as means±s.d. from two or three different donors, respectively. (**d**) Decreasing densities of ^51^Cr labeled gene-modified hMSCs were co-cultured with PBMCs in the presence or absence of CD33^+^ MOLM-13 cells at an e:t ratio of 5:1. After 20 h of co-incubation the specific hMSCs lysis was examined via chromium release assay. Data shown as mean±s.d. from two independent donors.

The capability of the MSC-released bsAb to redirect T lymphocytes in an antigen-dependent manner was evaluated with various AML cell lines expressing low (OCI-AML3), intermediate (U937) and high levels (MOLM-13) of CD33 antigen (Figure 2a). HEK293T cells served as a CD33 negative control cell line (Figure 2a). In the presence of the purified bsAb CD33–CD3, human T cells mediated target cell killing (MOLM-13 cells) in a concentration-dependent way (Figure 2b). In contrast, CD33^-^ HEK293T cells were not attacked by human T cells in the presence of the bsAb (Figure 2b). Next, retargeting of T cells to AML cells via *in situ* bsAb-releasing modified hMSCs was evaluated. In the presence of 48 h-cultured hMSC lines, 1x10^4^ CD3^+^ T cells were incubated with ^51^Cr labeled CD33^+^ AML cell lines at an effector-to-target cell (e:t) ratio of 5:1. After an incubation time of 20 h a large portion of target cells was lysed even at the lowest MSC seeding densitiy of 5 × 10^2^ cells/well (Figure 2c right). In fact, no relevant differences in specific target cell lysis between samples, containing different hMSC densities, was detected. Apparently, T cells can be redirected efficiently against tumor cells even at very low bsAb concentrations. In addition, the crosslinkage of T cells and target cells via hMSC-released bsAbs resulted in an effective killing of target cells at earlier time points, independently of the CD33 antigen density on the surface of cell lines (Figure 2c left). In contrast, no specific lysis was detected with the wt and vector control hMSC lines, confirming that the observed tumor cell killing was strictly dependent on the bsAb release. The determined killing efficacy of the bsAb CD33–CD3 is within the concentration range of published data.^2, 21, 23^ To further exclude any bsAb-dependent off-target effect on bsAb releasing MSCs, additional killing assays were performed. Therefore, 48 h-cultured, ^51^Cr labeled, gene-modified MSCs were incubated with PBMCs to resemble an *in vivo* situation, in which more complex cell populations are present. No off-target lysis of bsAb-expressing hMSCs occured after 20 h of co-culturing hMSCs and PBMCs in the presence or absence of CD33^+^ target cells (Figure 2d). However, parallel experiments performed under the same conditions showed efficient killing of CD33^+^ target cells similar to the data reported in Figure 2c. When isolated natural killer cells were incubated with CD33-expressing target cells both in the presence or absence of the purified bsAb no target cell lysis occured (Supplementary Figure S1e), ruling out any bsAb–Fc-receptor-based interactions. This is in accordance to the results obtained by flow cytometry, where also no binding of the bsAb to natural killer cells was detected (Supplementary Figure S1d).

### Improvement of T-cell-mediated tumor cell killing by a co-stimulatory ligand on the hMSC surface

**Figure 3:**
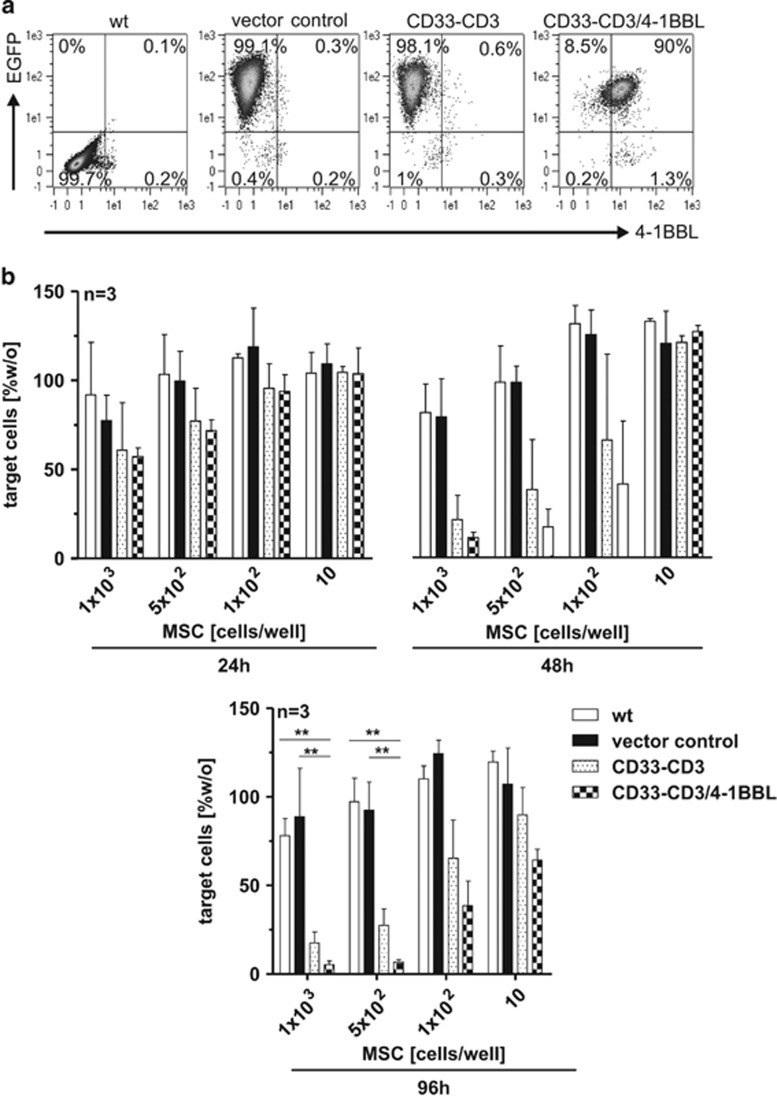
T-cell mediated tumor cell killing elicited by hMSC-produced anti-CD33-anti-CD3 recombinant antibody is enhanced by co-stimulation from 4-1BBL. (**a**) For the analysis of the 4-1BBL transgene expression hMSCs were stained with PE-conjugated anti-CD137L antibody and the surface expression of the immunoligand as well as intracellular EGFP signal correlating with bsAb CD33–CD3 expression were analyzed by flow cytometry. Positive cells are shown as percentages of all analyzed cells. Dead cells were excluded by propidium iodide counter-staining. Quadrant position was placed based on isotype control staining (not shown). (**b**) In a flow cytometry-based cytotoxicity assay eFluor670 proliferation dye-labeled CD33^+^ OCI-AML3 cells were incubated with pan T cells at an e:t ratio of 1:1 for 24 h, 48 h (upper) and 96 h (lower) in the presence or absence of gene-modified hMSCs seeded at limiting densities 48 h before the experiment. Target cell numbers counted at the indicated time points were normalized to the control sample with only target cells. Data represent the means±s.d. of three different donors. Statistical significance was determined using one-way analysis of variance with Bonferroni multiple comparison test. **P<0.01.

The anti-tumor effect of CD33 retargeted T cells can be reinforced by co-stimulation with 4-1BBL. 4-1BBL crosslinks with the 4-1BB molecule on activated T cells, potentiating selective tumor cell killing.^23^ To also test these findings for AML cells expressing low levels of the CD33 antigen, the bsAb-releasing hMSCs were further modified to co-express the 4-1BBL cell surface molecule (Figure 3a). Their immunotherapeutic effect was investigated by a flow cytometry-based cytotoxicity assay. HMSCs were seeded at very low concentrations and cultured for 48 h. Freshly isolated human T cells and eFluor670-labeled CD33^low^ OCI-AML3 target cells were co-incubated together with the modified hMSCs at an e:t ratio of 1:1, resembling the *in vivo* conditions of AML patients in acute blast crisis (with low T-cell numbers). Living target cells were quantified after 24 and 48 h by flow cytometry. As shown in Figure 3b (upper) T-cell mediated target cell lysis, triggered by the bsAb released from both immunotherapeutic hMSC lines (CD33–CD3 and CD33–CD3+4-1BBL), was delayed and could only be observed after 48 h. Co-stimulation of 4-1BBL presenting hMSCs did not significantly enhance the killing abilities of the bsAb redirected T cells. However, 4-1BB-mediated co-stimulation is mainly involved in late phases of immune activation.^23^ Hence, the percentage of living eFluor670-positive tumor cells was further analyzed after 96 h. The longer co-incubation ameliorated the T-cell response towards CD33^low^ target cells, leading to a more pronounced specific tumor cell killing even at the lowest hMSCs density of 10 MSCs/well (Figure 3b (lower)).

### Cytokine secretion and expansion of redirected T cells upon their crosslinkage with the bsAb and 4-1BBL

**Figure 4:**
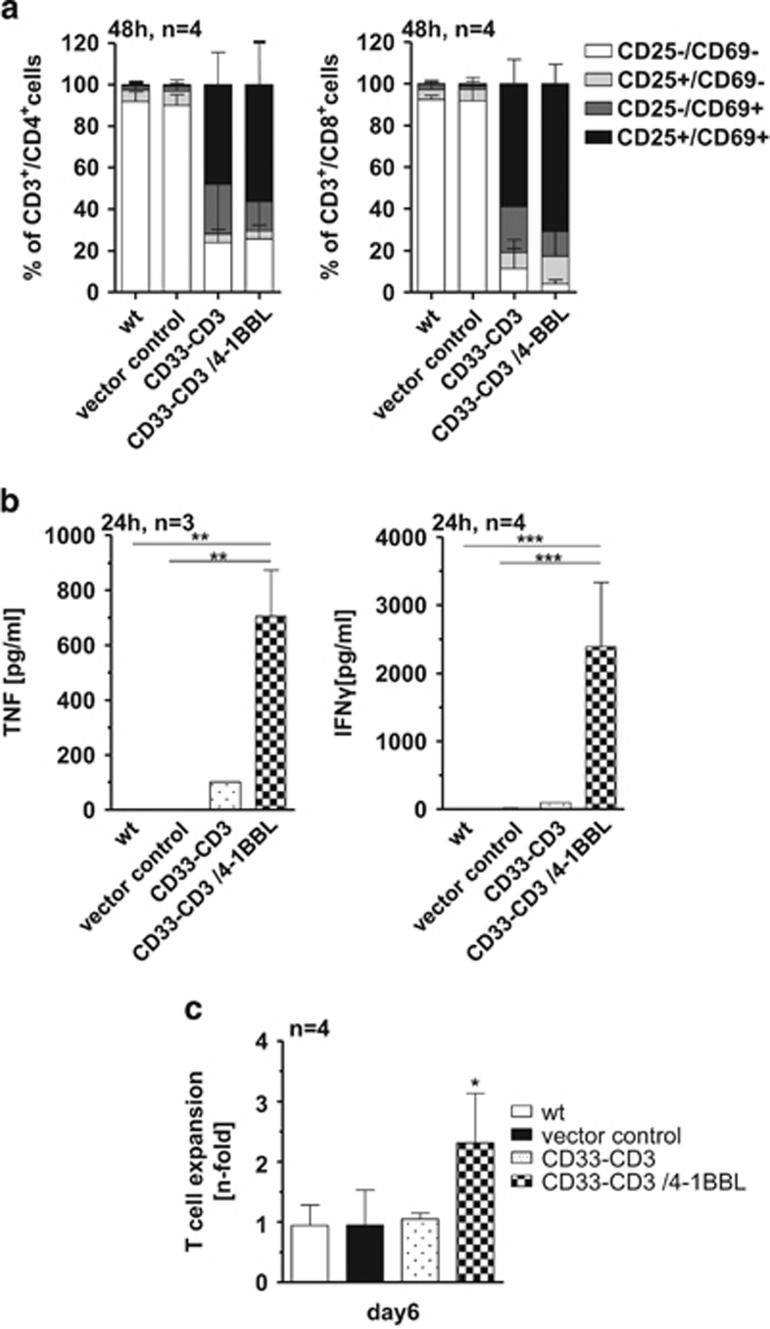
The presence of the co-stimulatory 4-1BBL signal markedly increases cytokine secretion and T-cell expansion. (**a**) Freshly isolated pan T cells were cultured together with eFluor670 proliferation dye-labeled CD33-expressing HEK293T cells at 5:1 e:t ratio in the presence of the genetically modified hMSCs. After 48 h of co-incubation, the activation status of redirected T cells was examined by staining for cell-surface expression of specific activation markers. Data represent the mean±s.d. of the average percentage of CD25^+^ and CD69^+^ T cells, CD4^+^ (left) or CD8^+^ T cells (right) of four independent donors. (**b**) Secretion of pro-inflammatory cytokines by T cells was determined after 24 h culture upon their crosslinkage with the transgenic CD33-expressing HEK293T cells via hMSC-produced CD33-CD3 bsAb in the presence or absence of the hMSC-presented co-stimulatory 4-1BBL. (**c**) After 6 days of co-cultivation with target cells T-cell counts were investigated and T-cell expansion was calculated as ratio of T-cell number at day 6 to T-cell number seeded at day 0. Data are shown as means±s.d. of three or four individual donors. Statistical significance was determined using one-way analysis of variance with Bonferroni multiple comparison test. *P<0.05; **P<0.01; ***P<0.001.

To verify the co-stimulating 4-1BBL/4-1BB effect in T-cell activation, pro-inflammatory cytokine releases and T-cell expansion were investigated. Transgenic CD33^+^ HEK293T cells, negative for T-cell co-stimulatory ligands, served as target cells. By using these instead of CD33^+^ native AML cell lines, additional T-cell stimulation, exerted by co-stimulatory molecules, could be excluded. Both CD4^+^ and CD8^+^ T cells upregulated the activation markers CD69 and CD25 in the presence of the two bsAb-producing hMSC lines at comparable levels (Figure 4a). However, when analyzing the amount of pro-inflammatory cytokines in the supernatants, marked differences were detected. Co-stimulation via 4-1BBL-expressing hMSCs significantly increased the levels of tumor necrosis factor-α (eightfold) and interferon-γ secretion (>10-fold; Figure 4b). T-cell crosslinkage with CD33^+^ HEK293T cells provoked lower interferon-γ and tumor necrosis factor-α releases. Nearly no cytokines were detectable in samples containing wt and vector control hMSCs (Figure 4b). In accordance with the increased cytokine secretion, crosslinkage with immunoligand expressing hMSCs induced stronger proliferation of bsAb-activated T cells. Thus, approximately two to threefold higher expansion rates of T cells were observed in the presence of the 4-1BBL presenting hMSCs. In contrast, absolute T-cell numbers were neither increased in the presence of the CD33–CD3-releasing hMSCs nor in the parental wt and vector control sub-lines (Figure 4c).

### Retargeting of autologous T cells towards AML blasts

**Figure 5:**
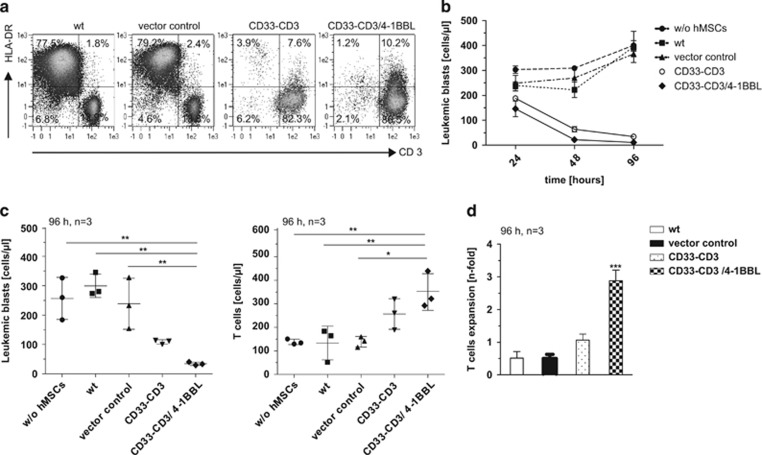
Antitumor effect of redirected AML patient-derived T cells against autologous AML blasts via hMSC-produced bsAb CD33–CD3 and surface presented 4-1BBL molecule. (**a**) 1 × 10^5^ AML patient-derived MNCs were cultured together with 48 h pre-seeded 1x10^4^ hMSCs. After 96 h of co-cultivation the percentages of surviving HLA-DR^+^ AML blasts and CD3^+^ T cells were determined respectively as proportions of all CD45^+^ cells by flow cytometry analysis. (**b**) Total AML blasts number after 24, 48 and 96 h of co-incubation was calculated. The average of surviving cells and the s.d. of triplets are shown for one representative donor out of three. (**c**) Total numbers of CD3^-^CD123^+^HLA-DR^+^CD45^+^ AML blasts (left) and CD3^+^CD123^-^HLA-DR^-^CD45^+^ T cells (right) after 96 h of co-cultivation with or without control/bsAb- and 4-1BBL-expressing hMSCs are reported for three independent donors. Numbers of each subpopulation were calculated according to their relative percentages as determined by staining for specific cell surface markers. (**d**) Absolute autologous T-cell number was measured after 96 h and overall expansion of the cells in the presence of hMSCs was determined. Data are presented as means±s.d. from three different donors. Statistical significance was determined using one-way analysis of variance with Bonferroni multiple comparison test. *P<0.05; **P<0.01; ***P<0.001.

To investigate their capability of redirecting autologous T cells towards AML blasts, CD33–CD3 bsAb-producing hMSCs were co-cultivated with AML patient-derived MNCs. Freshly isolated MNCs were phenotyped via immunostaining to measure the proportions of CD45+, CD123+ and HLA-DR+ AML blasts and CD45+, CD3+ T cells. MSC sub-lines were seeded 48 h before co-cultivation with patient-derived MNCs. Using specific surface markers, cells were stained after 24, 48 and 96 h of co-culture to quantify tumor cell killing and T-cell expansion by flow cytometry. Thereby, relative percentages and absolute cell numbers of leukemic cells and leukocyte populations were determined. Absolute leukemic blast numbers of a representative donor markedly decreased after 48 h of co-culture (Figure 5a and b). Within 96 h they were nearly completely eradicated. In the presence of control hMSCs, the number of leukemic blasts almost remained stable over time. AML blasts number decreased likewise in the presence of both bsAb-releasing hMSCs after 96 h of co-culture (Figure 5c left). The presence of the 4-1BB co-stimulus led to a pronounced (approximately threefold higher) autologous T-cell proliferation compared with other samples (Figure 5c right and 5d). Altogether, these results show that the continuous delivery of bsAb CD33–CD3, together with a constant stimulation of T cells by modified MSCs, improves specific killing of autologous AML blasts and increases patient-derived T-cell proliferation over time. Moreover, a co-stimulatory signal for bsAb-redirected and -activated T cells does not necessarily have to be mediated by interaction with the target cells. It can also be provided by independent cells.

### *In vivo* functionality of the bsAb-releasing hMSCs system

**Figure 6:**
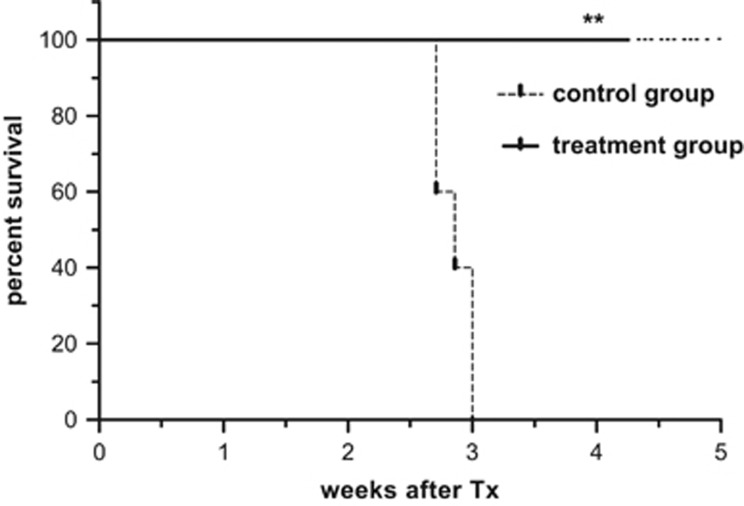
BsAb-releasing hMSCs prevent the establishment of AML in NOD/SCID IL2Rγ^−/−^ (NSG) mice. Kaplan–Meier survival analysis of NSG mice after intravenous injection of T cells and MOLM-13 AML cells at an e:t ratio of 5:1 together with vector control containing (dashed line) or bsAb-releasing (black line) hMSCs, indicated as control (*n*=5) and treatment group (*n*=4), respectively. A log-rank test was performed to determine the statistical significance in survival between the groups from ongoing experiment. **P<0.01.

The efficacy of the bsAb-secreting gene-modified cell system was investigated *in vivo* by co-injection of 24 h-pre-incubated MOLM-13 cells, T cells and bsAb CD33–CD3 releasing- (treatment group) or vector control containing- (control group) hMSC lines into NOD/SCID IL2Rγ^−/−^ mice. Mice of the control group developed signs of leukemic cell engraftment between the second and third week. They displayed weight loss, reduced locomotion, hunched posture and ruffled fur. In contrast, mice of the treatment group were protected from the disease (Figure 6). When the untreated control mice had to be killed, all treated mice were alive and did not show any signs of disease (significance of survival **P<0.01).

## Discussion

Mesenchymal stem cells posses a number of unique features, which make them highly attractive for an application as cellular weapons in anti-tumor therapy. Among these, is their potential to migrate into inflamed tissues and tumors. Inflamed tissues and tumors secrete cytokines and chemokines such as the (SDF-1α) stroma-derived factor one alpha, for which MSCs express the appropriate receptors on their surfaces. Thus, an MSC-based targeted cancer gene therapy can enhance the therapeutic efficacy, because MSCs can reach tumors and deliver therapeutic molecules in a concentrated fashion. Conventional AML therapy includes a combination of chemotherapy and hematopoietic stem cell transplantation, which results in high incidents of relapse and refractory diseases. Its limited ling term success makes the study of bsAb secreting MSCs attractive. Moreover, most clinical experience was gained with the CD33 as target antigen.

In this study, MSCs were gene-modified to secrete an autologous T-cell recruiting bsAb against AML blasts. CD33 is expressed on leukemic stem cells. Only CD33^+^ leukemic stem cells give rise to leukemia in mice. Most important, it has already been shown that T-cell activation against CD33-expressing cells, using the bsAb of this study, does not impair hematopoietic engraftment in NOD scid gamma knockout mice,^23^ but leads to efficient eradication of AML blasts both *in vitro* and *in vivo*.^21^

Using immortalized, human MSC line SCP-1 as a model, the feasibility of employing MSCs as cellular bsAb production machines was demonstrated. Gene-modified SCP-1 s secreted the bsAb in high concentrations even after prolonged in *vitro* culture. The average release of bsAb within 48 h was 8–70 pg/cell. In clinical trials the anti-CD3-anti-CD19 bsAb blinatumomab was administered as a continuous 24 h intravenous infusion at 15 μg/m^2^ per day for several weeks decreasing leukemic blasts below the detection level of minimal residual disease.^5^ Between 5.5 × 10^5–6^ gene-modified MSCs would be necessary to produce a comparable concentration of the bsAb CD33–CD3 (Figure 1e). Considering an average human cell weight of 1 × 10^−12^ kg,^29^ a feasible cell mass of about 5.5 mg would have to be transplanted into a patient for sufficient antibody production.

Although it has been described that MSCs may suppress activation of T cells,^30, 31^ this was not relevant in our experiments. Furthermore, bsAb-activated T cells did not attack modified MSCs, indicating their high specificity. Certainly, non-immunogenic properties of human MSCs may also contribute.^32, 33^

As the pro- or anti-tumorigenic effects of MSCs remain unclear,^34^ additional modifications of the MSCs, enhancing local anti-tumor inflammatory reactions, may be beneficial. One-way to augment anti-tumor effects of T cells was examined in our study. The additional T-cell stimulation, provided by tumor necrosis factor receptor signaling by 4-1BBL-expressing MSCs, resulted in a more pronounced and long-lasting T-cell-mediated response. Thus, T-cell proliferation potential and cytotoxic activity against AML blasts, expressing different levels of the CD33 antigen, were enhanced. The observation that targeting the 4-1BB pathway in cancer treatment ameliorates the elimination of tumor cells and prevents activation-induced cell death is in line with published data.^35, 36^ This advancement should be considered with respect to potential future *in vivo* use of gene-modified MSCs.

For developing MSCs as *in situ* producers of anti-cancer therapeuticals further, two ways for clinical application are possible. First, autologous, gene-modified MSCs could be injected intravenously into patients, promoting their migration to the tumor site and ensuring a local delivery of their payload. Second, as the effect of MSCs on tumor growth is still controversial,^37^ MSCs could be trapped in an artificial scaffold matrix and be transplanted subcutaneously close to a tumor site. In this way, a potentially supportive role of MSCs in tumor angiogenesis processes could be reduced. In addition, they could not escape from their confined, artificial environment. The delivery of the anti-cancer agent could be controlled and even stopped after tumor clearance by removing the scaffold.^38^

Altogether, our data show that continuous *in situ* delivery of bsAb CD33–CD3 by genetically modified hMSCs represents a promising alternative to the exogenous administration of short-lived immunoagents for the antigen-specific immunotherapy of AML patients. Moreover, the immunosuppressive potential of MSCs does not disqualify them as *in vivo* bsAb factory. Consequently, engineered MSCs could be a powerful tool in cancer therapy. As sources for sustained concentrations of highly efficient bsAbs, MSCs could help to achieve long-term anti-tumor responses and eventually tumor regressions.
