# Supplementary material for: Bispecific antibody releasing-mesenchymal stromal cell machinery for retargeting T cells towards acute myeloid leukemia blasts
Source: Blood Cancer J. 2015 Sep 18;5(9):e348–. doi: 10.1038/bcj.2015.73 (PMC4648523; doi:10.1038/bcj.2015.73)
Supplement: Supplementary Figure S1 [file bcj201573x1.pdf]

Supplementary Figure S1

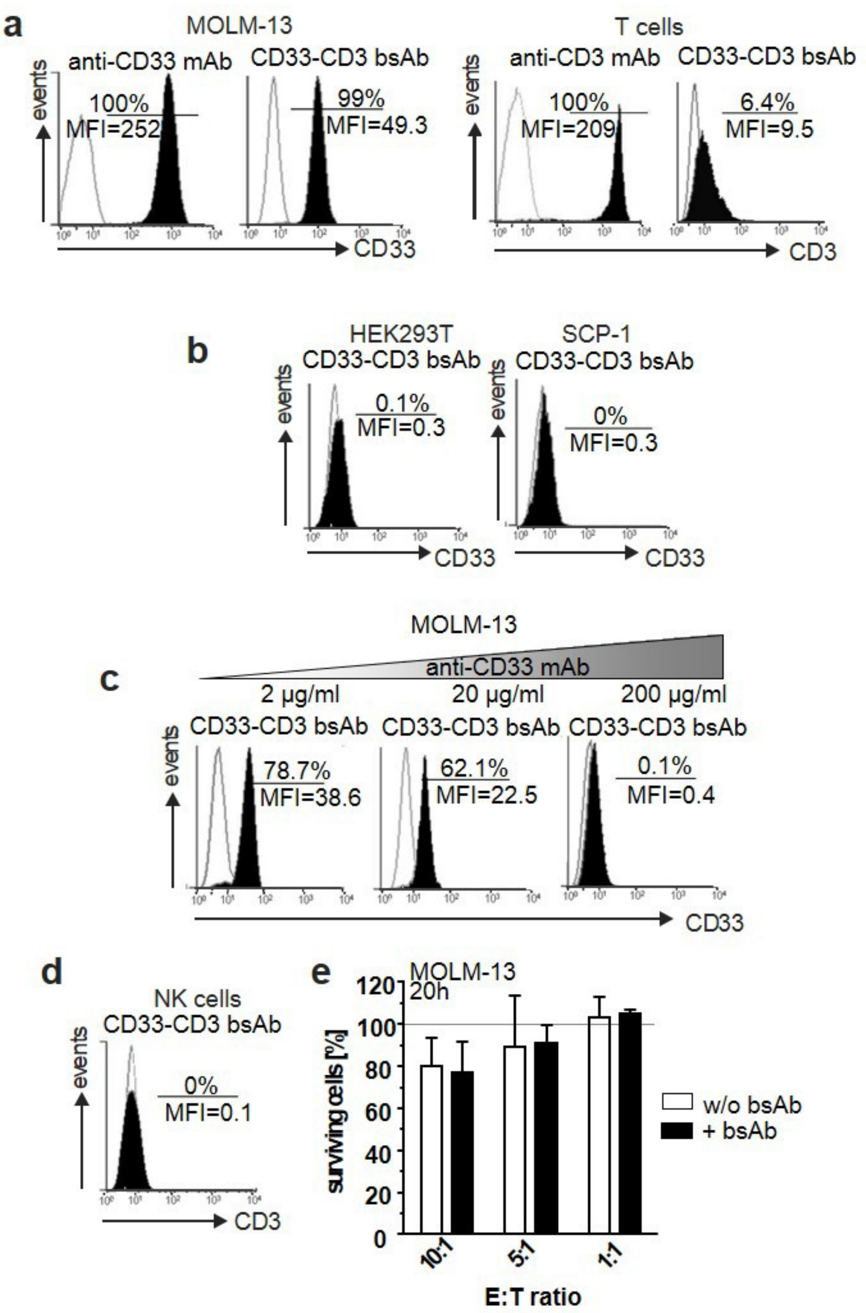

Supplementary Figure S1. Antigen binding properties of the hMSC-released recombinant protein. (**a**) CD33+ MOLM-13 cells (left panels) and PBMCs (right panels) were stained with either the maternal anti-CD33 and anti-CD3 mAbs following by a PE-conjugated anti-mouse IgG secondary Ab (black graphs) or the anti-CD33-anti-CD3 bsAb and a FITC-labeled anti-myc Ab (black graphs). (**b**) BsAb binding to CD33- HEK293T cells or SCP-1 wt cells was analyzed in parallel by flow cytometry after staining with the bsAb and the anti-myc/FITC Ab. (**c**) The accessibility of the bsAb to the CD33 antigen on MOLM-

13 cells after pretreatment with 2, 20 and 200 μg/ml respectively of the maternal anti-CD33 mAb is reported. (**d**) $CD3^-CD56^+CD16^+$ NK cells in isolated PBMCs were stained with the anti-CD33-anti-CD3 bsAb following by detection with a FITC-labeled anti-myc Ab (black graph). Mean fluorescence intensity (MFI) and percentage of positive cells are shown. Respective cells stained with the corresponding secondary antibodies served as negative controls (transparent graphs). (**e**) Percentage of surviving $CD33^+$ MOLM-13 cells detected by flow cytometry after 20 h of co-culture with NK cells at indicated effector-to-target cell ratios, in the presence or absence of 30 nM of the bsAb. Black line represents the percentage of surviving MOLM-13 cells detected in the absence of NK cells and set to 100%. Data shown as the mean ± SD of triplets of one representative donor.
